# Evaluation of Deep and Dynamic Proteomic Screening Strategies at Sub-50 Hz Scan Speed and without Robotic Sample Preparation

**DOI:** 10.1021/acs.jproteome.6c00312

**Published:** 2026-09-15

**Authors:** Bhavesh S. Parmar, Yuanyuan Liu, Parviz Ghezellou, Christian Münch

**Affiliations:** Institute of Molecular Systems Medicine, 9173 Goethe University, Frankfurt am Main 60528, Germany

**Keywords:** TMT, DIA, pSILAC, protein translation, protein degradation, Q Exactive HF, orbitrap, tribrid, unfolded protein response

## Abstract

Advances in ultrafast mass analyzer technology and procedural automation have enabled proteomic screening at the throughput of hundreds of proteomes per day. However, these approaches often require expensive instrumentation upgrades and robotic automation that remain inaccessible to many research laboratories and core facilities. In this study, we address the feasibility of scaling up proteomic screening capabilities with minimal upgrade cost by focusing on (a) strategies for nonautomated high-throughput sample preparation from 96-well cell culture, (b) data acquisition on sub-50 Hz scan speed hybrid and tribrid Orbitrap instruments, and (c) data analysis strategies for label-free and labeled proteomic screening. We find that the 96-well format STrap, in combination with C18 plates, provides the most robust throughput for a nonautomated sample preparation workflow. Furthermore, we show that for static proteomes, an isobaric tandem mass tag (TMT)-based multiplexing approach provides deeper and more precise proteome coverage, whereas data-independent acquisition (DIA) is more accurate, albeit with a reduced dynamic range and more missing values. Finally, we extend the optimized workflow to proteome turnover studies using pulsed stable isotope labeling by amino acids in cell culture (pSILAC), highlighting the key advantages and trade-offs of DIA and TMT data-dependent acquisition strategies for capturing protein translation.

## Graphical abstract

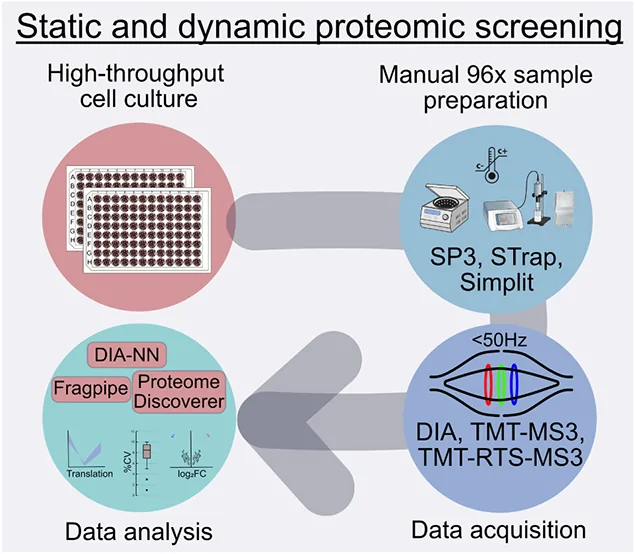

## Introduction

Over the past two decades, mass spectrometry (MS)-based proteomics has matured into a cornerstone technology for biological and biomedical research.^1–3^ This progression has been multifaceted, driven not only by advances in instrumentation and computational analysis but also by the expansion of training programs, shared core facilities, and standardized workflows that have broadened accessibility across academic, clinical, and industrial sectors. Routine bottom-up proteomics is now widely adopted, supported by robust and reproducible protocols and end-to-end commercial kits that lower experimental barriers and improve inter-laboratory reproducibility.^4^ Advanced liquid handling robots have further enabled automated high-throughput sample processing within relatively short time frames.^5–9^ On the acquisition side, recent innovations, including ion mobility separation, mass analyzers operating at scan rates exceeding 200 Hz,^10,11^ and robust liquid chromatography systems, have enabled analytical capacities approaching 500 samples per day.^12^ Notably, deep proteome coverage from complex biological matrices can now be attained with a sample runtime as brief as 15 min and 10$ of user cost.^13,14^ Consequently, fully unmanned proteomics workflows encompassing sample preparation and data acquisition have become technically feasible.^15^ Despite these advances, the practical accessibility of cutting-edge proteomics remains uneven. While the field increasingly favors time-of-flight-based instrumentation and greater end-to-end automation, a substantial proportion of laboratories—particularly those operating under resource limitations as of 2024—continue to rely on Quadrupole-Orbitrap (Q-OT) and Quadrupole-Orbitrap-Linear Ion Trap (Q-OT-LIT) instruments with moderate scan rates (<50 Hz)^16^ and non-automated methods.

In this study, we systematically evaluate sample preparation strategies, data acquisition modes, and data analysis approaches for both labeled and label-free proteomics, with the explicit goal of maximizing throughput and quantitative depth using Orbitrap-based instruments operating below 50 Hz scan rates and without reliance on automation. Our central objective is to define realistic, accessible proteomics workflows that can be implemented within standard cell and molecular biology laboratories, enabling high-throughput proteome measurements akin to those of cell screening platforms.

Specifically, we first assess high-throughput sample preparation strategies compatible with 96-well cell culture formats, comparing in-solution (adapted from Simplit^17^ hereafter named Simplit2), filter-based (STrap^18^), and on-bead aggregation-based (SP3 ^19^) enzymatic digestion. We then benchmark label-free quantitative data-independent acquisition (LFQ-DIA) and tandem mass tag-based data-dependent acquisition (TMT-DDA) across multiple Orbitrap platforms, including the Q Exactive HF, Orbitrap Fusion Lumos Tribrid, and Orbitrap Ascend Tribrid, evaluating achievable depth, throughput, and quantitative accuracy within a 24-hour experimental window. Lastly, we integrate 96-well cell culture with pulsed stable isotope labeling by amino acids in cell culture (pSILAC; 2SILAC and 3SILAC formats) to assess DIA- and TMT-based approaches for capturing temporal proteome dynamics in a medium- to high-throughput fashion. Collectively, this work provides a practical and adaptable framework for performing deep and dynamic proteomic screens using widely available Orbitrap instrumentation and non-automated workflows. The strategies outlined here may be transferable to diverse biological systems and multiple research contexts, such as clinical proteomics, chemoproteomics, and CRISPR- or siRNA-based functional proteomics screens. By aligning methodological rigor with infrastructural realities, this study aims to bridge the gap between state-of-the-art proteomic capabilities and their widespread, routine implementation.

## Materials and Methods

### Cell Culture and SILAC Labeling

HeLa cells were used for all the experiments and grown in RPMI 1640 or SILAC RPMI 1640 (Gibco—Product numbers 11875093 and 15477983) supplemented with 10% fetal bovine serum and appropriate amino acid isotopes (Silantes) (for SILAC). For sample preparation optimization, 20,000 HeLa cells/well were seeded in a 96-well cell culture plate (Sarstedt) and grown overnight in 100 μL of medium. The medium was decanted, and adherent cells were gently washed 3× with 200 μL phosphate-buffered saline (PBS) and frozen with 75 μL of lysis buffer (see below). For dynamic proteome analysis with thapsigargin/DMSO treatment, 20,000 cells/well were seeded in a 96-well plate and grown overnight in 100 μL of SILAC Light (K0, R0) medium. Following that, the light medium was decanted, and the cells were rinsed with 200 μL PBS once and treated with either 1 μM thapsigargin or dimethyl sulfoxide (DMSO) in a heavy (K8, R10) medium for 2 h. The cells were then washed 3× with PBS, and one batch (plate) was frozen for the 2SILAC experiment in 75 μL of lysis buffer. Another plate was further incubated with an SILAC intermediate (K4, R6) medium for 2 h, rinsed 3× with PBS, and frozen in 75 μL of lysis buffer for the 3SILAC experiment.

### High-Throughput Sample Preparation from 96-Well Cell Cultures

For STrap and SP3, the lysis buffer contained 5% sodium dodecyl sulfate (SDS), 5 mM tris­(2-carboxyethyl)­phosphine (TCEP), 25 mM chloroacetamide (CAA), and 1× protease inhibitor cocktail (Roche cOmplete EDTA-free—11836170001) in 100 mM triethylammonium bicarbonate (TEAB). For Simplit2, the lysis buffer contained 2% sodium deoxycholate (SDC), 5 mM TCEP, 25 mM CAA, and 1× protease inhibitor cocktail in 100 mM TEAB. For all samples described in Figures 1 and 3, plates were thawed by snap-heating at 95 °C for 10 min in a ThermoMixer C (Eppendorf) and spun at 1,500 × g. Following that, the samples were sonicated using an 8-horn probe sonicator (Qsonica, part no. 4599) connected to an ultrasonic processor (Sonics Vibra Cell VCX130) at 40% amplitude for 2 min with 1 s on/1 s off pulses. The samples were reduced with 1 μL of 500 mM TCEP and alkylated with 5 μL of 500 mM CAA, both for 20 min at RT. For the Simplit2 protocol, 0.2 μg of Trypsin+Lys-C mix (1:1) (Promega V5111 and VA1170) was added directly to the plate, sealed with a silicon mat between the plate and the lid, and incubated overnight at 37 °C. The digested peptides were acidified with 200 μL of 99% isopropanol/1% trifluoroacetic acid and loaded onto a styrene divinylbenzene reverse phase sulfonated (SDB-RPS) plate (Affinisep AttractSPE microelution μ96W-RPS-M.T.1.1) attached to a 1.2 mL deep well plate and spun at 600 × g for 2 min. The peptides were washed once with 200 μL of 99% isopropanol/1% trifluoroacetic acid and 200 μL of 2% acetonitrile/0.1% trifluoroacetic acid by spinning at 1,500 × g for 1 min. The cleaned peptides were eluted in 80% acetonitrile/5% ammonia and dried in a vacuum concentrator. For the STrap plate, the samples were acidified with 5 μL of 25% phosphoric acid, and the lysate was transferred into a 1.2 mL deep-well plate (Abgene AB1127). 700 μL of 90% methanol, 100 mM TEAB was added to the lysate and loaded onto STrap (ProtiFi—96-well microplate) in 2 batches of 390 μL and spun at 1,500 × g for 1 min with a 1.2 mL deep-well plate underneath. The samples were washed 3× with 200 μL 90% methanol, 100 mM TEAB (Sigma-Aldrich T7408) by centrifugation at 1500 × g for 1 min each. Following that, 0.2 μg of Trypsin+Lys-C mix (1:1) in 125 μL of 100 mM TEAB was added to the filter and incubated overnight at 37 °C on a fresh 1.2 mL deep-well plate. The digested peptides were eluted from STrap along with 80 μL of 100 mM TEAB first, then with 80 μL of 0.2% trifluoroacetic acid, and lastly with 80 μLof 50% acetonitrile/0.2% trifluoroacetic acid. The peptides were dried in a SpeedVac before being resuspended in 100 μL of 0.2% trifluoroacetic acid for C18 cleanup. For SP3, the lysate was transferred to a 1 mL LoBind plate (Eppendorf—0030504208) and 5 μL of 50 μg/μL prewashed beads (1:1 Cytiva SpeedBead Magnetic Carboxylate—6515,4515) were added, followed by 100 μL of ethanol and vortex for 5 min at 1200 rpm. The plate was set on a magnetic rack (LifeSep Z740158) for 10 min to allow the beads to separate, and the liquid was decanted using a multichannel pipette. This process was then repeated 3× with 100 μL of 80% acetonitrile. The beads with protein aggregates were then resuspended in 100 μL of 0.2 μg Trypsin+Lys-C mix, briefly sonicated to detach the beads, and incubated on a ThermoMixer at 37 °C overnight with intermittent shaking at 1,200 rpm for 2 min every 1 h. The digested SP3 peptides were acidified to 0.2% trifluoroacetic acid and spun at 1,500 × g for 2 min before being set on a magnetic rack for 10 min and collecting the acidified peptides for C18 cleanup. All C18 plate (Affinisep—AttractSPE microelution μ96W-C18-M.T.1.1) spins were performed at 600 × g for 3 min with a 1 mL deep-well plate attached underneath. First, C18 was washed with 200 μL of 50% acetonitrile/0.2% trifluoroacetic acid and equilibrated 3× with 200 μL of 0.2% trifluoroacetic acid. The STrap and SP3 samples were loaded and washed three times with 200 μL of 0.2% trifluoroacetic acid, followed by elution with 50% acetonitrile/0.2% trifluoroacetic acid and drying in a vacuum concentrator. The samples were then resuspended in either 100 mM TEAB for the microBCA peptide assay or 2% acetonitrile/0.1% trifluoroacetic acid for LC-MS/MS analysis.

**Figure 1:**
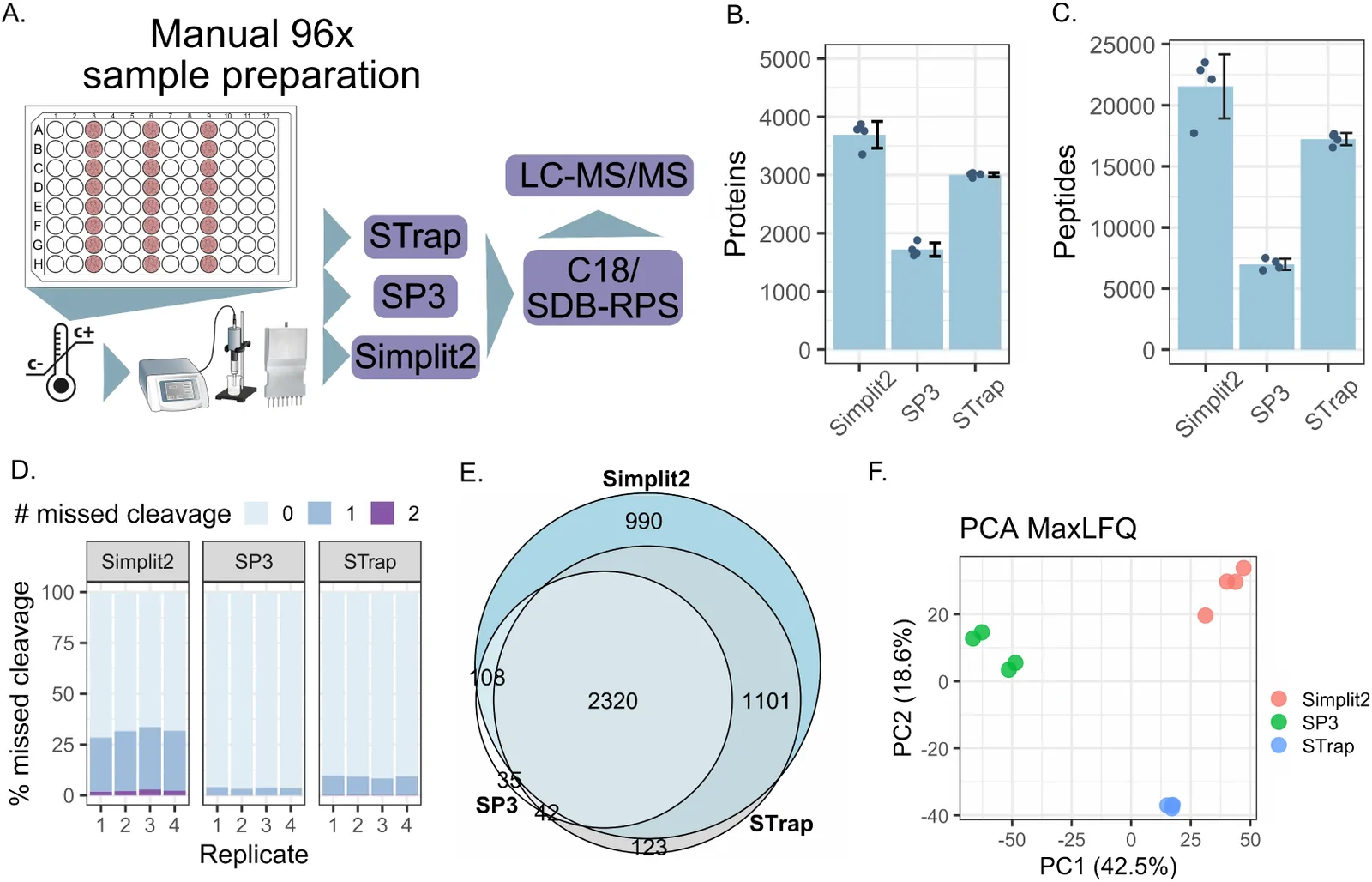
Comparison of high-throughput, non-automated sample preparation methods for cells grown in a 96-well plate. A. Experimental scheme of cell culture and sample preparation methods compared for manual processing. 20,000 cells were seeded and grown overnight, followed by freeze–thaw lysis, homogenization using multi-probe sonication, and digestion using STrap, SP3, or Simplit2 protocol, followed by C18 or SDB:RPS cleanup. B. Proteins and C. peptides identified across replicates from 125 ng of digest using the DDA-LFQ method. D. Proportion of tryptic missed cleavages across the three methods. E. Overlap of proteins identified across the three methods. F. Principal component analysis of MaxLFQ intensities per replicate showing intersample variance within each compared method.

### Three-Species Proteome Benchmarking Mixtures

Commercially purchased peptide digests for bacteria (*Escherichia coli*, MassPREP-Waters 186003196), yeast (*Saccharomyces cerevisiae*, Promega V7461), and human (K562, Promega V6951) were resuspended in 10% acetonitrile to a concentration of 2 μg/μL and pooled at different human:bacteria:yeast ratios (Ctrl—65:15:20; Mix1—65:30:5; Mix2—65:20:15) in triplicate. For LFQ, the pooled digests were diluted to 1% acetonitrile/0.2% trifluoroacetic acid and stage-tipped (Empore, 3M, 2215-C18), as with C18 plates, but with centrifugation at 1,500 × g. For TMT labeling, samples were diluted to achieve 100 mM 3-[4-(2-hydroxyethyl)­piperazin-1-yl]­propane-1-sulfonic acid (EPPS) in 10% acetonitrile, labeled with TMT10-plex, cleaned up using a C18 Sep-Pak cartridge (tC18, 50 mg; Waters WAT054960), and fractionated by high-pH C18 reversed-phase chromatography (see below).

### pSILAC Experimental Design for Dynamic Proteome Measurements

The dynamic SILAC plates were processed in the same fashion following the above-mentioned STrap protocol, followed by C18 cleanup in the 96-well format. For DIA analyses, the samples were resuspended in 15 μL of 2% acetonitrile and 0.1% trifluoroacetic acid, and 3 μL was injected. For TMT, samples were resuspended in 20 μL of 100 mM EPPS and 10% acetonitrile, cleaned up, fractionated, and run on Ascend and Lumos.

### Isobaric Labeling with TMT and TMTpro

For the 3 species mix, 10 μg per sample was labeled with TMT10 in the following order of replicates per condition: Ctrl (127N, 127C, 128N), Mix1 (128C, 129N, 129C), and Mix2 (130N, 130C, 131N). For dynamic 2SILAC and 3SILAC combined with TMT, the samples were labeled with TMTpro such that all replicates per condition received the same tag (N or C): DMSO (127N–134N), thapsigargin (126C–133C). A dried 5 mg TMT stock was resuspended in 250 μL of 100% acetonitrile, and 1 μL (for ∼10 μg of the peptide) was added to the above-mentioned samples and incubated for 1 h at 24 °C. Following that, the reaction was quenched with 0.5% hydroxylamine (final concentration) for 15 min at 24 °C. 1 μL of each TMT channel was pooled to confirm the labeling efficiency via LC-MS/MS. Following confirmation of >99% labeling, the channel volumes were calculated based on the median S/N and pooled together.

### High-pH Reversed-Phase Peptide Fractionation

The pooled TMT channels were acidified to 0.2% trifluoroacetic acid and cleaned with a C18 Sep-Pak cartridge (tC18, 50 mg, Waters) with 3× 2% or 5% acetonitrile/0.1% trifluoroacetic acid (for TMT11 and TMTpro, respectively), eluted with 60% acetonitrile, and dried in a vacuum concentrator. The dried TMT11 and TMTpro samples were resuspended in 40 μL 5 mM ammonium bicarbonate/5% acetonitrile and sonicated in a water bath for 1 min, and the concentration was measured using microBCA. Sample concentration was adjusted to 1.5 μg/μL, and 45 μg of the sample was used for high-pH reversed-phase fractionation on an Ultimate 3000 HPLC system (Thermo Fisher Scientific) equipped with a C18 column (XSelect CSH, 1 mm × 150 mm, 3.5 μm particle size; Waters SKU186005251). Peptides were separated using a multistep gradient from 3 to 60% Solvent B (5 mM ammonium bicarbonate in 80% acetonitrile) over 65 min at a flow rate of 30 μL/min. Eluting peptides were collected every 43 seconds starting at minute 2 for 69 min into a total of 96 fractions, which were concatenated into 24 or 12 fractions. Pooled fractions were dried in a vacuum concentrator and resuspended in 2% acetonitrile/0.1% trifluoroacetic acid for LC-MS analysis.

### LC-MS/MS Data Acquisition

Detailed acquisition parameters for all DDA and DIA methods are provided in (Supplementary Table S1 Sheets 1 and 2). All LC-MS analyses were performed using nanoLC systems (Easy-nLC 1200 or Vanquish Neo; Thermo Fisher Scientific) operated at a flow rate of 400 nL/min and coupled to hybrid Orbitrap mass spectrometers (Q Exactive HF, Orbitrap Fusion Lumos, and Orbitrap Ascend). Mobile phases consisted of Solvent A (0.1% formic acid in water) and Solvent B (80% acetonitrile with 0.1% formic acid). Samples were loaded at 950 bar pressure using 6 μL of Solvent A onto an in-house-packed 35 cm fused-silica column (75 μm inner diameter) packed with 1.9 μm Reprosil-Pur C18 particles (Dr. Maisch, r119.b9). For experiments shown in Figure 1, data acquisition was performed on a Q Exactive HF coupled to an Easy-nLC 1200. Approximately 125 ng of peptides (quantified using a microBCA assay) were injected and separated over a 90 min gradient consisting of 4–20% B in 60 min, 30–40% B in 20 min, and 40–60% B in 5 min, followed by 9 min of column wash at 95% B. For three-species mixture experiments using TMT-MS3 acquisition on an Easy-nLC 1200 coupled to an Orbitrap Fusion Lumos, approximately 500 ng per fraction was loaded and separated using a 90 min gradient (5–10% B in 3 min, 10–28% B in 72 min, 28–40% B in 15 min, and 40–60% B in 5 min, followed by 9 min of column wash at 95% B). For three-species mixture experiments with TMT-MS2 and TMT-MS3 [real-time search (RTS) and real-time search closeout (RTScl)] on the Vanquish Neo coupled to an Orbitrap Ascend, approximately 250 ng per fraction was injected and separated over a 90 min gradient (7–28% B in 70 min, 28–40% B in 13 min, 40–60% B in 5 min, followed by 6 min of column wash at 95% B). For real-time search (RTS) experiments, SPS-MS3 triggering was enabled using a custom three-species FASTA database containing one gene per protein sequence for each organism, downloaded from UniProt. RTS searches were performed either with (RTScl) or without (RTS) peptide/protein closeout, with the closeout threshold set to two peptides per protein. RTS parameters included TMT at the peptide N-terminus and lysine as fixed modifications and methionine oxidation as a variable modification, an XCorr cutoff of 1, and a deltaCn of 0.1 at 5 ppm. For DIA experiments, 250 ng (Orbitrap Ascend) and 500 ng (Q Exactive HF) of peptides were injected and separated using 45, 60, 90, or 120 min gradients with optimized isolation window schemes and instrument-specific acquisition parameters (Supplementary Table S1). For DIA with pSILAC-labeled samples (2SILAC and 3SILAC, *n* = 4 biological replicates per condition), 500 ng was loaded on the column, and the same 120 min gradient was used on the Q Exactive HF and Lumos (both online with Easy-nLC 1200), with MS1 and DIA scans acquired at 120,000 and 30,000 resolution (at *m/z* 200), respectively (Supplementary Table S1). For pSILAC-TMTpro experiments, data were acquired using MS3 on the Orbitrap Fusion Lumos and MS3-RTS on the Orbitrap Ascend (*n* = 8 biological replicates per condition). Identical acquisition settings were used on both instruments, with the Targeted Mass Difference (TMD) node enabled to detect either two or three isotopic states (2SILAC and 3SILAC, respectively), with the key difference being MS3 resolution (45K or 50K) and maxIT (91 ms or 86 ms), respectively, and 250 ng of the sample was loaded on the column. The fractions were separated over a 120 min gradient (7–25% Solvent B ramp in 90 min, followed by 40% in 30 min and 60% Solvent B in 5 min). RTS for pSILAC-TMTpro experiments was set by adding heavy and intermediate lysine and arginine SILAC masses as variable modifications, together with methionine oxidation, two variable modifications allowed per peptide, and a human UniProt FASTA database (one gene per sequence). All other RTS parameters were identical to those used for the three-species experiments.

### Proteomic Data Processing and Statistical Analysis

DDA data were analyzed using Proteome Discoverer (v3.2) or FragPipe v23.0, while DIA data were processed using DIA-NN v2.2. Across all search engines, cysteine carbamidomethylation (+57.02146 Da) was specified as a fixed modification, while protein N-terminal acetylation (+42.0106 Da) and methionine oxidation (+15.9949 Da) were included as variable modifications. For TMT experiments, TMT labeling of peptide N-terminal and lysine residues was specified using mass shifts of +304.20715 Da (TMTpro) and +229.16293 Da (TMT classic). For the sample preparation comparison experiments, “Basic search” workflow in FragPipe was used with default parameters and match-between-runs (MBR) disabled. All TMT experiments were searched on FragPipe with default settings for MS2 or MS3 reporter ion quantification and searched against either a concatenated three-species FASTA database or the human UniProt FASTA database (one gene per sequence). For pSILAC-TMTpro experiments, the default SILAC workflows in FragPipe were adapted to include TMT and SILAC+TMT masses as variable modifications, and “Run Isobaric Quant” was enabled in the Quant (Isobaric) tab. For both three-species and pSILAC DIA searches on DIA-NN, first, a spectral library was generated with default parameters using either the three-species concatenated FASTA or the human UniProt FASTA database, and then, .raw files were subsequently searched against the resulting predicted spectral library (predicted.speclib file). For pSILAC DIA analysis, additionally, —fixed-mod SILAC,0.0,KR,label —lib-fixed-mod SILAC —channels SILAC,L,KR,0:0; SILAC,H,KR,8.014199:10.008269 —original-mods —channel-run-norm for 2SILAC, and —channels SILAC,L,KR,0:0; SILAC,M,KR,4.025107:6.020129;SILAC,H,KR,8.014199:10.008269 —original-mods —channel-run-norm for 3SILAC were added into “Additional options”. The resulting report.parquet files were processed in RStudio using the DIA-NN R package. All DIA results were filtered for Q-value, PG.Q-value, Lib.Q-value, Lib.PG.Q-value, and channel.Q-value (for SILAC), all set to <0.01, along with Quantity.Quality >0.8 before protein rollup. The number of data points per chromatographic peak was estimated by dividing the retention time width of precursor signals by the maximum cycle time for each acquisition method. For pSILAC DIA, the precursors from report.parquet from DIA-NN were separated based on channel assignment, and for pSILAC-TMT, PSMs were separated based on peptide modification. FragPipe PSM.txt outputs were filtered for Purity >0.5 and Peptide probability >0.9, and the lowest 5% of PSM intensities were removed prior to protein summarization using parsimony-based inference. PSM output from Proteome Discoverer was filtered for isolation interference (>0.5), SPS match percentage (> 60), and PSM uniqueness, before summing signal-to-noise ratios for protein rollup. For all TMT results, the filtered PSM intensities were normalized to the total reporter ion intensity per TMT channel before protein summarization, and for DIA, PG.MaxLFQ intensities were used for downstream analysis. Only proteins quantified across all samples were considered, and differential expression was evaluated using Limma (v3.66.0) with default lmFit and eBayes functions; gene ontology enrichment was performed using clusterProfiler (v4.16.0) with the enrichGO function and all identified proteins/experiment as “background universe”, and visualization was executed via ggplot2 (v4.0.2) in RStudio/2025.05.1 with R v4.5.0 and above. All the analysis codes along with input files are available on Zenodo (DOI: 10.5281/zenodo.18925014).

## Results

### Comparison of High-Throughput and Non-Automated Sample Preparation Methods for 96-Well Cell Culture

We systematically evaluated three non-automated, high-throughput sample preparation workflows compatible with 96-well cell culture formats: Simplit2 (an adaptation of the Simplit protocol^17^), SP3,^19^ and STrap^18^ (Figure 1A). These methods differ substantially in reagent cost, hands-on complexity, digestion efficiency, and the time required for sample preparation, as investigated in detail by Varnavides et al.^4^ Cells in individual wells were lysed by freeze-thawing, sonicated with a multi-horn probe, and digested with the aforementioned methods, followed by cleanup with C18 (for SP3 and STrap) or SDB-RPS stage tips (for Simplit2). Evaluating sample measurements across replicates, Simplit2 and STrap yielded higher numbers of protein and peptide identifications relative to manual SP3 processing (Figure 1B and C). Importantly, Simplit2 produced a higher proportion of missed cleavages compared to SP3 and STrap (Figure 1D). Although STrap mini plates are typically recommended for protein input between 100 and 300 μg (by the vendor), we evaluated their performance at lower inputs to assess suitability for 96-well cell culture experiments. Using HeLa lysate inputs ranging from 5 to 100 μg, we observed consistent protein and peptide identification numbers across all inputs, with only a marginal increase in variability at 5 μg (Figure S1A-C). However, for manual SP3 in the 96-well format, we observed a drastic increase in variability below 25 μg input, with fewer proteins and peptides identified, possibly due to sample loss during processing (Figure S1D-F). Moreover, STrap coupled with C18 cleanup provided a higher peptide recovery compared with Simplit2 combined with SDB-RPS or SP3 combined with C18 cleanup (Figure S1G). The identified proteins were consistent across the three methods (Figure 1E) with Simplit2 producing relatively larger peptides (Figure S1H) owing to higher missed cleavage proportion (Figure 1D). Importantly, quantitative reproducibility across replicates was highest for STrap, which exhibited the lowest coefficient of variation relative to Simplit2 and SP3 (Figure 1F, Figure S1I). Dilution of HeLa lysate (5–25 μg) digested with manual SP3 in the plate format produced fewer identifications below 25 μg, suggesting that the poor performance observed in 96-well cell culture SP3 plate digests may be due to lower input amounts. We further assessed the compatibility of STrap and Simplit2 with isobaric labeling (as shown in the original Simplit publication^17^). While unfractionated TMTpro samples show ∼80% overlap in proteins measured with both methods (Figure S1J), the results are in concordance with our LFQ observations, with less inter-sample variation observed with STrap compared with Simplit2 (Figure S1K-L). Overall, these results demonstrate that both STrap and Simplit2 are well suited for non-automated 96-well proteomic sample preparation workflows, whereas SP3 may require further optimization for manual 96-well plate handling, especially for <10 μg inputs (Figure S1M). Between STrap and Simplit2, the former offers better digestion efficiency and quantitative reproducibility, whereas the latter is relatively simpler and more economical for high-throughput proteomic screens at the expense of increased inter-sample variability and a higher proportion of missed cleavages (Figure S1M).

### Comparison of Data Acquisition Strategies for Deep Global Proteomics

To benchmark data acquisition strategies under constrained scan-rate conditions, we analyzed three-species peptide mixtures spanning a broad dynamic range of peptide abundances using either label-free data-independent acquisition (DIA) or tandem mass tag (TMT)-based data-dependent acquisition (DDA) workflows. Peptide digests derived from bacteria, yeast, and human proteomes were combined in defined ratios (Figure 2A) to generate control (Ctrl), Mix1, and Mix2 sample mixes (*n* = 3 replicates/sample mix). For label-free DIA, each mixture was injected individually and analyzed using four different chromatographic gradient lengths on both the Orbitrap Ascend and Q Exactive HF mass spectrometers in order to estimate the sample throughput and proteome depth achievable for both instrumental setups. We selected fixed isolation-window schemes that resolved the spectral complexity, preserved at least six median data points per chromatographic peak, and avoided trapezoidal quantitation^20^ (Figure S2A). The data point per precursor were estimated using precursors RT.Start and RT.Stop, the maximum attainable cycle time, and median full-width at half-maximum (FWHM) values for each LC-MS configuration (Figure S2B–C). For all the DIA methods, the distribution of the Quantity.Quality metric for the majority of precursors was consistently above the threshold of 0.80 (Figure S2D). For isobaric TMT-based DDA, samples were separated by high-pH reversed-phase fractionation and analyzed either on an Orbitrap Lumos using MS3 acquisition with 24 fractions (90 min gradients) or on an Orbitrap Ascend using 12 fractions (90 min gradients) with MS2, MS3 with real-time search (MS3RTS), or MS3RTS with closeout, limiting MS3 sampling to two peptides per protein per fraction (MS3RTScl) (Figure 2A). Detailed acquisition parameters and instrument settings are provided in the Materials and Methods section and Table S1.

**Figure 2:**
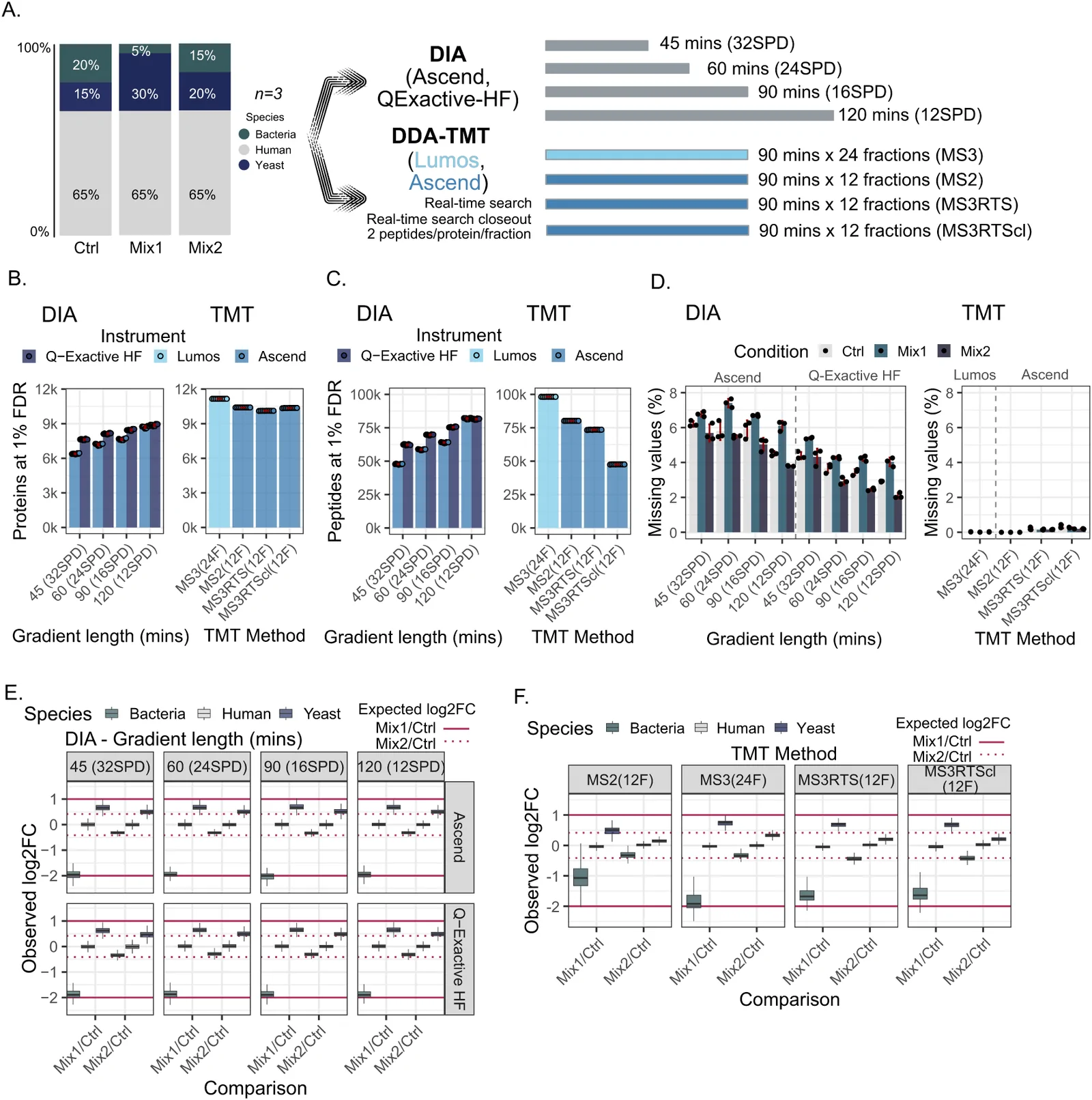
Comparison of data acquisition strategies for deep global proteomics. A. Tryptic peptide digests of yeast and bacteria were mixed with human background in three different ratios (namely Ctrl, Mix1, and Mix2) to create a dynamic range of differences of 2-fold (yeast—Mix1 vs Ctrl), 4-fold (bacteria—Mix1 vs Ctrl), or 1.33-fold (yeast and bacteria—Mix2 vs Ctrl). The samples were run individually (*n* = 3 technical replicates per sample) either as DIA on Q Exactive HF and Ascend with 4 different gradient lengths amounting to 4 levels of throughput or labeled with TMT, fractionated into 24 or 12 fractions, and run over a 90 min gradient with SPS-MS3 (on Lumos—light blue) or MS2, real-time search (RTS)-triggered SPS-MS3, and RTS-triggered SPS-MS3 with a 2 peptides/protein/fraction close-out option on Ascend (dark blue). B. Proteins and C. peptides quantified at 1% FDR for all the compared DIA and TMT methods, with dots indicating identifications per replicate. D. Percentage of missing values per method, with dots corresponding to sample type (Ctrl, Mix1, Mix2) and error bars showing standard deviation across all 9 samples. E. Boxplot showing the distribution of log2 fold change for each comparison (Mix1/Ctrl and Mix2/Ctrl) across all methods compared, with the black line within each box indicating the median observed log_2_ fold change and red lines at expected log_2_ fold change (solid for Mix1/Ctrl and dotted for Mix2/Ctrl).

Under these conditions, fractionated TMT-DDA consistently yielded greater proteome depth than single-shot, library-free DIA. Ranked by depth, TMT-MS3 with extensive fractionation (24 fractions) performed best, followed by MS2 (12 fractions), MS3RTScl (12 fractions), and MS3RTS (12 fractions) (Figure 2B,C). With equivalent acquisition time, the three TMT approaches using 12 × 90 min fractions quantified >10,000 proteins (mean peptide counts per TMT channel ≈ 80,180, 73,519 and 47,569 for MS2, MS3RTS, and MS3RTScl, respectively), compared to <9,000 proteins with 81,937 and 81,509 peptides for DIA on the Ascend and Q Exactive HF, respectively (Figure 2B,C). TMT-MS3 without RTS on the Lumos achieved the deepest coverage (>11,158 proteins and 98,171 peptides) but required twice the acquisition time. As expected, the quantified proteins and peptides progressively declined with shorter gradient lengths in DIA, with a more pronounced loss observed for the Vanquish Neo-Orbitrap Ascend configuration relative to Easy-nLC-Q Exactive HF, consistent with the generation of sharper chromatographic peaks by Vanquish Neo, which places greater demands on MS duty cycle and chromatographic sampling under compressed gradient conditions (Figure 2B,C). Missing values in DIA ranged from roughly 2 to 7% and were elevated for mixtures with a larger dynamic range (Mix1, Ctrl) relative to mixtures with smaller inter-species abundance differences (Mix2) (Figure 2D) with a consistent <5% median coefficient of variation (%CV) across all the compared methods (Figure S2E-F). Based on the number of proteins quantified per minute of acquisition time, 45 min (32SPD) DIA is the most economical; however, TMT-based methods could be further leveraged with increased plexing such as TMTpro18 or TMTpro32 instead of TMT11 (classic) utilized in this experiment (Figure S2G).

With regard to quantitative accuracy, DIA and MS3 outperformed MS2 and MS3RTS methods in the higher fold-change range with median log_2_FC close to the expected value of −2 for the bacterial proteome, whereas all methods were consistently inaccurate at the difference of log_2_FC +1 (for the yeast proteome), with the highest relative accuracy achieved by TMT-MS3 (Figure 2E,F). As expected, TMT with MS2 was the least accurate in all regards due to ratio compression^21^ (Figure 2F). The deviation in accuracy appears to be dependent on summed protein abundances (MaxLFQ for DIA and signal-to-noise (S/N) for TMT), with consistent dispersion observed at lower values across all compared methods and gradient lengths (Figure S3A-B). In the lower FC range, where log_2_FC ± 0.415 was expected, all TMT methods with 12 fractions (MS2, MS3RTS, MS3RTScl) struggled with accuracy (Figure 2F). This observation agrees with previous studies that employed ten TMT fractions versus ten DIA runs,^22^ suggesting that accuracy in TMT experiments might be dependent on first dimension fractionation. However, this phenomenon could also be instrument-dependent as TMT-MS3RTS on the Orbitrap Ascend has previously been shown to incur a slight deviation in accuracy.^23^ DIA, on the other hand, performed consistently across varying gradient lengths irrespective of the instrument setup, suggesting that DIA is capable of robust quantification even in the log_2_FC ± 0.415 range, independent of elution complexity (Figure 2E), specifically with >6 average data points/peak (Figure S2C) as reported previously.^20^

Next, we compared TMT-MS3RTS datasets processed with Proteome Discoverer 3.2 and FragPipe version 23.0 (Figure S3C-E). A similar comparison has previously been conducted, differing only slightly in parameters and in the use of an additional Chimerys module.^24,25^ Our findings are broadly consistent with the earlier report,^25^ showing approximately 95% overlap in protein quantification between the two analysis tools (Figure S3C), although the correlation of measured fold changes was below 90% (Figure S3D). FragPipe-derived intensities exhibited reduced accuracy relative to Proteome Discoverer’s signal-to-noise–based and TMT-purity-corrected quantification, particularly for differential abundance ≤ twofold change (log_2_FC ± 1) (Figure S3E) suggesting that TMT purity correction might be essential to accurate quantification. We further assessed whether TMT correction or the presence of empty channels in the Proteome Discoverer quantification workflow affected the results. Although both corrected and uncorrected approaches showed high correlation, omission of TMT correction introduced a systematic slope shift (y = 0.91x + 0.0072) and decreased quantitative accuracy (Figure S3F), whereas the presence of empty channels in the analysis did not seem to affect the overall quantitative accuracy (Figure S3G). Together, our results provide evidence for deeper proteome coverage and quantification achieved with TMT-based methods as compared to LFQ-DIA within our setup, with similar quantitative accuracy achieved by LFQ-DIA and TMT-MS3. Moreover, the accuracy for MS3 methods may be dependent on TMT correction and the number of fractions, with less fractions and uncorrected TMT providing less quantitative accuracy. Overall, our findings support the use of TMT-MS3 (with or without RTS) for achieving deep and accurate proteome coverage on tribrid instruments, whereas DIA-based analyses may be better suited for hybrid systems where TMT reporter ions cannot be resolved in MS^n^ or corrected posthoc. The DIA approach, however, offers considerable speed and cost benefits when maximum depth and data completeness is not a primary requirement.

### Evaluation of Dynamic Proteomic Screening with pSILAC-TMT and pSILAC-DIA

To assess whether our optimized digestion from 96-well cell culture and acquisition strategies are compatible with dynamic proteome analyses, we designed a pulsed stable isotope labeling (pSILAC) experiment using 2SILAC and 3SILAC labeling schemes to monitor protein synthesis. Cells were treated with thapsigargin, and its effects on protein translation (2SILAC) as well as recovery post-treatment (3SILAC) were measured, as compared to DMSO-treated controls (Figure 3A). Thapsigargin is an inhibitor of the sarco-endoplasmic reticulum Ca­(2+)-ATPase SERCA, induces the unfolded protein response (UPR), and causes translational attenuation.^26^

**Figure 3:**
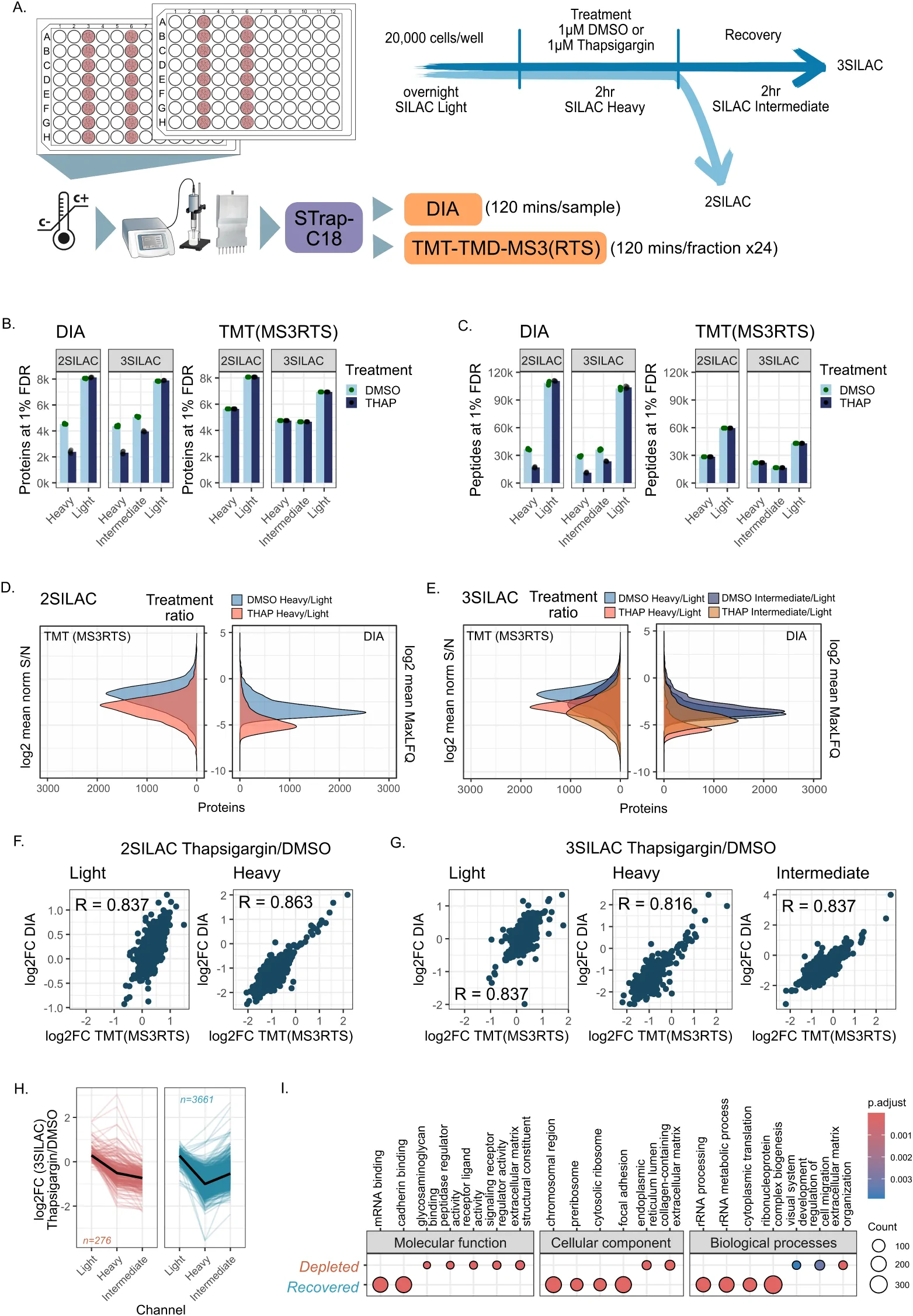
Evaluation of dynamic proteomic screening with pSILAC-TMT and pSILAC-DIA. A. Experimental scheme for dynamic proteomics with pSILAC coupled treatment with DMSO or thapsigargin (2SILAC) and recovery post-treatment (3SILAC). 20,000 cells/well were grown overnight, treated for 2 h, and recovered for 2 h. The samples were processed with the established STrap protocol and then either run as a TMTpro experiment (*n* = 8 per condition, 24 fractions × 120 min) with the targeted mass-difference node activated (TMD) and a real time search-assisted MS3 scan or run as a DIA experiment (*n* = 4 per condition × 120 min). B. Number of proteins and C. peptides quantified across both DMSO (dark blue) and thapsigargin (THAP) (light blue) treated cells in the 2SILAC and 3SILAC setup across all included SILAC channels. D. Log2 distribution of the mean ratio of heavy/light protein abundance (S/N for TMT­(MS3RTS) and MaxLFQ for DIA) for 2SILAC, and E. heavy/light and intermediate/light protein abundance for 3SILAC for DMSO- and thapsigargin (THAP)-treated cells. F. Correlation of the estimated log2 fold change (thapsigargin/DMSO) for light and heavy proteome pools for 2SILAC, and G. light, heavy, and intermediate for 3SILAC between DIA and TMT (MS3RTS) methods. H. Log2 fold change (thapsigargin/DMSO) of proteins quantified with all 3 isotopes in the 3SILAC experiment, showing basal levels (light), treatment-induced translatome (heavy), and post-treatment recovery of proteins (intermediate). The left panel (brown) shows proteins that remain depleted after removal of thapsigargin, and right (turquoise) shows proteins that recover after abolishing thapsigargin treatment by changing growth media. I. Gene ontology annotation of the depleted and recovered pools of proteins, showing their molecular function, cellular localization, and biological process associations, from the 3SILAC (TMT-MS3RTS) experiment.

The cells were processed with the previously established STrap protocol (this study, Figure S4A), and the performance of the different measurement approaches was compared by analysis using DIA on the Q Exactive HF and Lumos, or TMT-based MS3 acquisition with targeted mass difference (TMD)-enabled isotope selection,^27^ with or without RTS, on the Ascend and Lumos, respectively (120 min × 24 fractions; Figure 3A). Comparison of DIA and TMT-MS3RTS revealed distinct performance characteristics across SILAC plexes. DIA yielded consistent protein and peptide identifications across both 2SILAC and 3SILAC experiments, whereas TMT-MS3RTS exhibited a reduction in identification depth when moving from 2SILAC to 3SILAC (Figure 3B,C). Of importance, while the proteins quantified with DIA in the light channel expectedly remained unchanged across both treatments, a significantly lower number of proteins and peptides were quantified in thapsigargin-treated samples, reflecting the profound effect on protein synthesis within cells and consequently leading to missing values for quantitative statistics in DIA measurements. Conversely, TMT-based methods consistently identified all isotopes for both treatments with the 2SILAC and 3SILAC setup, albeit with reduced log_2_ fold changes (Figure S4B), making TMT-MS3RTS more amenable to quantitative differential abundance analysis without the need for imputation. This is also reflected in the ratio of heavy and intermediate SILAC-labeled protein abundances relative to their light pretreatment counterparts, wherein TMT ratios show a more symmetrical Gaussian distribution across the dynamic range, and DIA MaxLFQ ratios show a sharp truncation of peaks on the lower end for both DMSO and thapsigargin (Figure 3D,E). Moreover, the relative %CV for TMT-MS3RTS was significantly better for 2SILAC and marginally better for 3SILAC in comparison to DIA (Figure S4C). For TMT-MS3 and DIA on the Lumos (Figure S4D-E), the trend was similar to that observed with DIA on the Q Exactive HF and TMT (MS3RTS) on the Ascend; however, the number of proteins and peptides are slightly lower for the same acquisition time as compared to TMT-MS3RTS on the Ascend or DIA on the Q Exactive HF. This agrees with a previous in-depth comparison of the Lumos and the Q Exactive HF-X, wherein Orbitrap MS^n^ acquisition on the Lumos underperformed.^28^

More than 80% correlation was observed in the differential protein abundance profiles between thapsigargin- and DMSO-treated cells when comparing DIA and TMT (MS3RTS) analyses (Figure 3F-G) across all isotopes. In line with previous observations, proteins labeled with a heavy isotope were consistently downregulated following thapsigargin treatment,^29–31^ whereas the majority of intermediate-isotope-labeled proteins (3,661/4,656) showed recovery toward basal levels during the post-treatment phase (Figure 3H). These were mainly involved in translation/ribosomal machinery and RNA processing (Figure 3I). A small proportion of proteins (*n* = 276) remained depleted even after the removal of thapsigargin and were found to be mainly localized in the extracellular matrix, secretory vesicles, and the endoplasmic reticulum lumen (Figure 3H, I).

Furthermore, we adapted the search settings for SILAC-TMT on FragPipe 23.0 with a custom roll-up of proteins from PSM.txt files and found strong agreement among proteins identified and quantified with Proteome Discoverer 3.2 (Figure S4F-G). Interestingly, while most proteins across all three isotopes were quantified with both search engines, FragPipe seemed to have a bias for higher heavy and intermediate identifications, whereas Proteome Discoverer had a bias for light isotope-labeled proteins (Figure S4F). The isotope error correction feature within MSFragger-FragPipe 23.0, which is absent in Proteome Discoverer 3.2, is likely the cause of this discrepancy. Together, our results suggest a deeper coverage of a dynamic proteomic screen incorporating 2SILAC with RTS-enabled MS3, whereas DIA achieves consistent coverage independent of the number of SILAC channels.

## Discussion

In this study, we evaluate an accessible framework for deep and dynamic proteomic screening from 96-well cell culture formats using nonautomated workflows and Orbitrap-based instrumentation operating below 50 Hz scan rates. By systematically benchmarking sample preparation strategies, acquisition modes, and data analysis approaches, we demonstrate that robust proteome depth and quantitative performance can be achieved with manual sample preparation from 96-well cell culture plates and sub-50 Hz scan speed mass spectrometers. These findings address a practical gap between state-of-the-art proteomics capabilities and the infrastructural realities of many laboratories that do not have access to cutting-edge technology available.

Plate-based cell culture assays are routinely applied within various contexts in cell biology and functional genomics. Yet, the limited protein yield per well often necessitates robotic liquid handling or a positive pressure platform to ensure robust and reproducible sample preparation. Here, we showed that comparable throughput and reproducibility could be achieved using standard laboratory equipment, such as a multichannel pipette, an 8-horn sonication adapter, deep-well plates, and a common plate centrifuge. We chose to test the three sample preparation methods based on the previous knowledge of higher peptide identifications relative to other methods,^4^ with STrap plates being the most expensive (approximately +€5/sample additional material cost) and Simplit2 being the least (no additional material cost). Among the evaluated workflows, both STrap with C18 cleanup and Simplit2 combined with SDB-RPS enabled reliable processing of 96-well cell culture samples without automation, with the latter being a more economical alternative, while the former being the most robust for label-free and isobaric tagging-based quantitative proteomics (Figure 1A–F, Figure S1A-L). Although the Simplit2 workflow yielded a lower peptide yield, increased inter-sample variability, and a higher proportion of missed cleavages relative to STrap, several factors could plausibly mitigate these limitations. Reducing the concentration of sodium deoxycholate in the lysate, employing a higher-efficiency proteolytic enzyme, or transferring lysates from cell-culture plates to vessels optimized for proteomics could each improve digestion efficiency and reduce losses due to surface absorption. As an alternative low-cost strategy, *n*-dodecyl-β-maltoside (DDM), a mass spectrometry-compatible detergent previously applied in single-cell proteomics,^32,33^ may offer improved solubilization with reduced interference. While SP3 in the 96-well plate format is a viable low-cost method and applicable for a wide range of detergent cleanup, it demands longer time due to intermittent vortexing and magnetic separation, and there is a potential risk of sample loss from multiple sample-pipette tip contacts during manual aspiration, as reported previously,^34^ suggesting that it may be better suited for robotic liquid handling, especially for input amounts <10 μg from 96-well cell culture plates. In addition, low-cost alternatives for STrap using DNA purification miniprep filters have been reported to achieve relatively better identifications,^35^ although their suitability for 96-well plate or higher throughput applications remains to be systematically evaluated. Of critical importance here are the plates used for cell culture as there are various matrix options available for aiding cell adherence, which might, however, contribute to background contaminants or adsorptive losses. In this study, we therefore employed standard tissue-culture-treated polystyrene plates to minimize such confounding effects.

In our benchmarking with three-species peptide mixtures, DIA delivered consistently accurate quantification on both LC-MS platforms across active gradients from 120 to 45 min (12–32 samples per day; Figure S3A, Figure 2E), although identification depth declined progressively as throughput increased. The sharper loss of identifications observed for the Vanquish Neo-Orbitrap Ascend configuration at the shortest gradients could be due to three factors i.e., (i) lower automatic gain control (AGC) settings or shorter injection times for Ascend DIA scans can reduce the number of ions sampled per scan and thereby sensitivity; (ii) operating the Ascend at reduced MS2 resolving power to preserve cycle time sacrifices fragment separation and may lower identifications in crowded spectra; and (iii) narrower chromatography peaks on Vanquish Neo compared to Easy-LC at the same flow rate of 400nL/min. While we focused on maintaining comparable average numbers of data points per chromatographic peak across platforms, performance of the Vanquish Neo-Orbitrap Ascend configuration at shorter gradients could likely be further improved by optimizing the Vanquish Neo flow rate, Ascend DIA scan AGC, and resolution. Irrespective of instrument tuning, we observed that missingness in DIA is strongly driven by sample dynamic range (Figures 2D, 3B,C). Consequently, both experimental design and downstream data processing should explicitly account for this behavior, for example, through appropriate sample loading, conservative false discovery rate thresholds, sparsity-aware statistical frameworks, judicious use of imputation, or analysis platforms that explicitly model DIA missingness, such as Spectronaut.^36^

TMT workflows using MS3 (TMT-MS3, TMT-MS3RTS, and TMT-MS3RTS with close-out) delivered the deepest proteome coverage with precise quantitation in our evaluation and achieved near-complete data matrices. We did not see a substantial increase in proteins quantified with RTS close-out methods (2.45%), suggesting that the detection threshold likely plateaued with 12 fractions. However, the close-out method might be advantageous when using fewer than 12 fractions or shorter gradients for TMT-multiplexed samples as it selectively limits MS3 triggering, thereby reducing redundant MS3 sampling of highly abundant proteins and reallocating duty cycle to lower-abundance species relative to the RTS method without close out (Figure 2C). These advantages of TMT can be further leveraged by incorporating higher-plex reagents (e.g., 32-plex),^37^ which allow for a relatively higher throughput with an RTS method (12 fractions of 90 min each = 1080 min total) per day as compared to 45 min DIA (32SPD = 1440 min total) analyses. A key practical consideration for TMT-based workflows is the cost of reagents, labeling-efficiency checks, fractionation infrastructure, and the associated bench time imperative to reconcile their advantages. Additionally, proteome depth in TMT experiments may be compromised with increasing pSILAC plexing in dynamic proteomics, likely reflecting stochastic sampling effects arising from the expanded peptide isotope pool. By contrast, DIA remains largely agnostic to an increase in isotopes if the fragment ions are effectively resolved (Figure 3B,C, Figure S4D). However, the limitation of DIA dynamic range persists even in pSILAC, and a lower number of peptides were identified in thapsigargin-treated samples, reflecting translation attenuation upon thapsigargin treatment and recovery thereafter (Figure 3B,C, F,G, Figure S4D). A potential concern in DIA-based SILAC experiments is that isotopic pairs may be split across adjacent isolation windows and potentially cause distortion in the SILAC ratio due to differential peak sampling, as noted previously.^38^ We reasoned that this potential distortion would not affect turnover measurements in pSILAC experiments as isotopes can be treated as separate protein pools and relied on channel-specific confidence metrics (channel Q-value) implemented in DIA-NN v2.2 to separate the precursor isotopes prior to protein roll-up. Consistent with prior benchmarking, such an approach has been shown to preserve detection sensitivity and quantitative accuracy across large isotopic imbalances, including ratios down to 1:100 isotopic difference,^39^ supporting the applicability of DIA to dynamic SILAC analyses. On tribrid instruments, the ion trap can be utilized for accelerated DIA acquisition with narrower isolation windows and resonant CID-type fragmentation to achieve more data points per peak and deeper coverage.^40^ However, resonant CID in an ion trap is prone to lower mass accuracy relative to Orbitrap HCD,^41^ and HCD fragmentation generates a richer population of singly charged y-ions,^42^ particularly informative for resolving SILAC isotopes, especially when coisolated and cofragmented within the same DIA windows; thus, we favored Orbitrap-based HCD acquisition for isotope-resolved DIA in this study. Although DIA achieved a slightly lower proteome depth than fractionated TMT workflows under the current instrumental setup, it required substantially less sample preparation time and incurred lower consumable costs. TMT reporter-ion quantitation can also be performed at the MS2 level on hybrid Orbitrap instruments (for example, the Q Exactive HF), which, in principle, may increase throughput and deliver comparable proteome coverage with fewer fractions or runs. However, as shown in Figure 2F, MS2-level TMT quantitation is inherently affected by reporter-ion interference arising from co-isolated peptides, leading to substantial ratio distortion that typically requires posthoc correction via computational approaches or experimental strategies, such as baseline channel incorporation.^21,31,43,44^

For TMT data analysis, we used Proteome Discoverer and compared results to the freely available FragPipe (academic license). Both pipelines produced highly overlapping protein lists, but we observed small systematic differences in quantitative accuracy that we attribute primarily to TMT purity correction and differing handling of isotope errors. In our pSILAC-TMT data, FragPipe’s isotope-error correction improved identification and quantification of heavy/intermediate channels relative to Proteome Discoverer, suggesting that it is advantageous for dynamic-pSILAC with TMT. It will be worthwhile to test other freely available analysis tools that support TMT correction (for example, Comet^45^ and MaxQuant^46^), especially for SILAC-TMT with a ground truth dataset to more fully assess performance for dynamic-TMT designs. For DIA, we relied on DIA-NN 2.2, especially for its channel-aware scoring for plexDIA/pSILAC measurements and its improved FDR estimation compared to previous versions. Nevertheless, users should consider entrapment-based FDR estimation (as described previously^47^) especially in older DIA-NN versions and/or filter the report.parquet file for Q-value, PG.Q-value, Lib.Q-value, Lib.PG.Q-value, and channel.Q-value (for SILAC) all set to <0.01 along with Quantity.Quality >0.8. The stringency of the Quantity.Quality filter is based on instrument performance and peak integration within DIA-NN, which in our case was well above 0.7 for the majority of precursors (Figure S3D); however, it is crucial to filter low scored precursors, especially precursors close to 0, for reliable quantitation. A detailed guide on processing different SILAC experiments by Frankenfield et al.^48^ and the approach from this manuscript can serve as a primer for handling pSILAC DIA data analyzed with DIA-NN.

In conclusion, we present an accessible and cost-effective framework for deep and dynamic proteomic screening in a 96-well cell culture plate, based on nonautomated, low-cost sample preparation workflows, widely deployed hybrid and tribrid Orbitrap instruments, and open-access analysis tools. By systematically comparing isobaric TMT- and DIA-based quantitation strategies for both global and pSILAC proteomes, we demonstrated the temporal resolution that can be achieved in understanding cellular trafficking and biological processes in a proteomic screening format. This framework can be further adapted for chemo- and geno-proteomics to achieve biologically important insights on older generations of instruments and still compete meaningfully in the race of ultrafasts.

### Limitations of This Study

While we have tried to benchmark sample preparation and data acquisition approaches geared toward proteomics screening applications on sub-50 Hz instruments, there are certain limitations in this study, mainly due to accessibility of relevant equipment/resources. Firstly, in our hands, SP3 did not perform well below 25 μg and was excluded from further testing with TMT. Additionally, there are more manual sample preparation approaches, as discussed, that may perform more efficiently and economically but remain to be systematically evaluated and compared against advanced robotic liquid handling setups. Secondly, TMT-based approaches require additional experimental steps, incur reagent costs, and rely on fractionation and specialized mass spectrometers to function robustly. Moreover, data integration and imputation across multiple plexes can be challenging and impact the overall throughput and turnaround time of a TMT-based screen. Thirdly, on the data acquisition side, while we have provided evidence of benchmarking and performance on three sub-50 Hz instrumental setups, a true comparison between these instruments is fundamentally confounded by massive differences between the instruments and the online LC. In addition, it was not possible to compare the data described to cutting-edge high-speed instruments.

## Supplementary Material





## Data Availability

The .raw files and processed files from Proteome Discoverer, FragPipe, and DIA-NN can be found on ProteomeXchange under identifiers PXD073367 and PXD073468. Search results and R codes for data processing and visualization can be found on Zenodo (DOI: 10.5281/zenodo.18925014).

## Notes

Optimization of high-throughput, non-automated sample preparation methods and TMT multiplexing of peptides for cells grown in a 96-well plate (Figure S1); DIA data acquisition parameters and quality statistics (Figure S2); qualitative and quantitative statistics of DIA and TMT and a comparison of the TMT data analysis strategy (Figure S3); comparison of DIA and TMT methods for pSILAC (Figure S4) (PDF)
Instrument parameters for data acquisition with DDA-LFQ,DDA-TMT and DIA for data presented in this study (Sheet 1); DIA isolation windows and gradient information for data in Figure 2 (Sheet 2) (Table S1) (XLSX)
